# Storing Information Electrically in Human Skin

**DOI:** 10.2478/joeb-2021-0010

**Published:** 2021-11-29

**Authors:** Oliver Pabst, Øystein Magnus Sørebø, Karoline Sjøen Andersen, Erlend Lemva Ousdal, Sean William Bråthen, Badi Ur Rehman, Haiatullah Gholami, Zhijian Zhou, Koki Takahashi, Diriba Tasfaye Dumesso, Mellie Merete Livingston, Wesley Julian Lodewijk, Stian Sæther, Alireza Eskandari Turk, Peter Louis Uller

**Affiliations:** 1 Department of Physics, University of Oslo, Oslo, Norway

**Keywords:** Bioimpedance, human skin, memristor, information storage, non-linear electrical measurements

## Abstract

Human skin has been classified as a non-volatile memristor and it is shown that information can be stored within for at least three minutes. Here we investigate whether it is possible to store information up to 20 minutes. Furthermore, we investigate whether the information can be based on four different states, not just two (binary). We stored the information into the skin of the forehead of the test subjects under three different electrodes, which allows in principle for 64 different combinations (3 electrodes, 4 states) and one can think of numbers on the base of four. For this experiment, we decided on the numbers 123_4_ and 302_4_ (that correspond to numbers 27 and 50 in the decimal system). Writing of the different states was done by the application of DC voltage pulses that cause electro-osmosis in the sweat ducts (nonlinear electrical measurements). Based on our results, we were not able to distinguish between four different states. However, we can show that binary information storage in human skin is possible for up to 20 minutes.

## Introduction

The memristor (memory resistor) has been found to be the forth passive electrical component [1], and it connects voltage and current via the state-dependents Ohms’ law


(1) v=M(x)i,

with *M(**x**)* as the memristance (state dependent resistance) and ***x*** as the vector of the internal state variables with


(2) dx/dt=f(x,i)⋅i.

Research interest in the memristor increased when a first realization was found in 2008 [2] (based on titanium dioxide). Memristors based on different materials like tantalum oxide [3], and zinc oxide [4] have been demonstrated, as well as, biological memristors like slime moulds [5] and apples [6], for example. However, in case of the apples the shown memristive properties may not origin from the apple itself but rather from chemical reactions on the electrodes [7].

In this study we focus on human skin, which is a memristor, as well [8]. There are actually two different memristor types that can be found in human skin [9]. One is based on electro-osmosis in the sweat ducts [10] (sweat duct memristor) and the other one is based on the non-linear electrical properties of the stratum corneum [11], the outer skin layer which is surrounding the sweat ducts. Both mechanisms are based on the movement of ions. Sweat contains ions and dependent on the polarity of the applied electrical stimulus, sweat is pulled towards the skin surface or deeper skin layers. Some ions may also move through the ionic pathways in the stratum corneum [12] and changes in the state-dependent resistance may be related to corresponding changes in temperature of the stratum corneum, which acts as a negative temperature coefficient thermistor [9]. It has been shown that the overall human skin memristor can be classified as generic, and non-volatile [9, 14].

Information storage is possible in non-volatile memristors and the information can potentially be analog and not just binary [15]. It has been shown that the conductance of human skin can be manipulated, for example, by direct current (DC) voltage pulses and that increased or decreased conductance states can still be read after three minutes [14].

In this study, we wanted to further investigate the information storage properties of human skin. We studied the small signal conductance after applied DC pulses for a longer period (20 minutes) instead of 3 minutes. Furthermore, we defined 4 distinct states of the skin conductance: 0 as the state after negative DC pulses were applied, state 1 as the baseline state (without applying a voltage), 2 as the state after positive DC voltage pulse, and 3 as the state after positive DC voltage pulses for a longer time than for state 2. We placed 3 electrodes at the forehead beside each other and stored one of the four conductance states underneath each of the electrodes. Four defined states and three skin sites lead to 64 combinations (43) that could potentially be stored in human skin by our setup. We choose numbers 123_4_ and 302_4_ for our experiment and we chose randomly for each measurement run which of both numbers were stored. We did the experiment twice on each subject (before and after physical exercise) to see whether information can be deleted by physical exercise and whether a new information can be written afterwards.

## Materials and methods

### Subjects recruitment, ethical approval, informed consent

The measurements were performed on 17 healthy test subjects (14 male, 3 female, mean age 29 years, SD=7.6 years) at the University of Oslo in October/November 2019. The research related to human use has been complied with all relevant national regulations, institutional policies and in accordance with the tenets of the Helsinki Declaration. Informed consent has been obtained from all individuals included in this study.

### Instrumentation, electrical safety and electrodes

The same instrumentation as in [14, 16] was used. A data acquisition card (DAQ) (type USB-6356 from National instruments) enabled the application of a constant voltage and simultaneous reading. The DAQ was connected to a personal computer; all powered by an international medical isolation device (IMEDe 1000 from Noratel AG, Germany), to ensure physical separation between test subjects and the mains. The software that controls the DAQ was written in NI LabVIEW (version 2019).

**Fig. 1:**
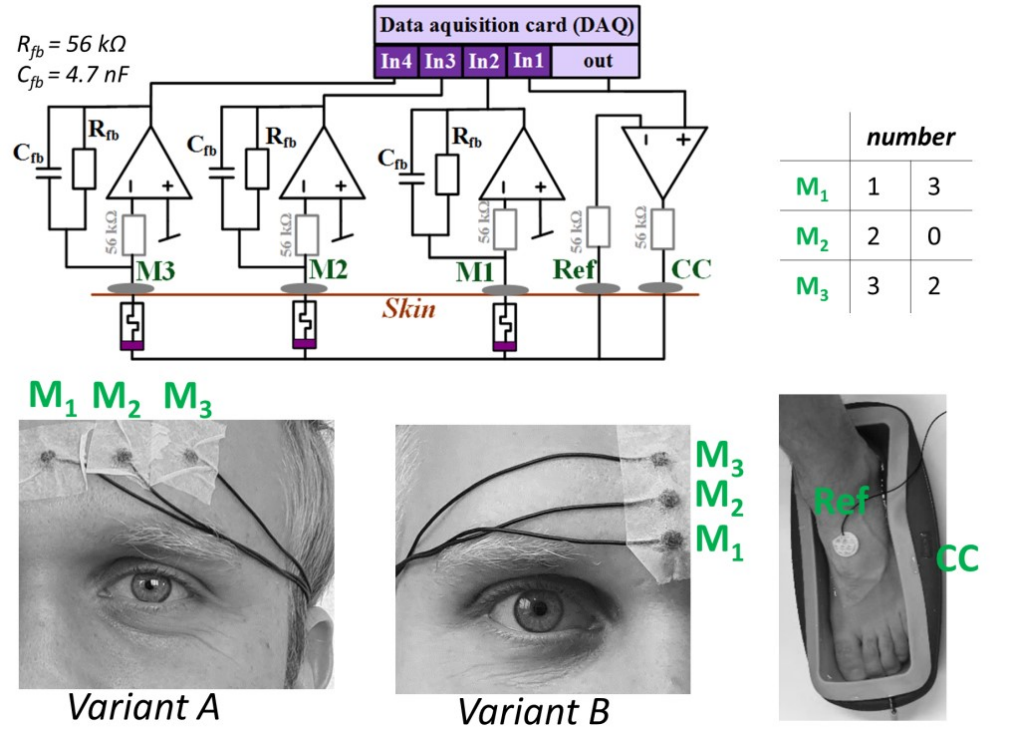
Schematic of the used instrumentation (same schematic as presented in [9] under Creative Commons Attribution 4.0 International License), electrode placement and the two chosen numbers and the corresponding states (0,1,2,3) under each electrode. The used three electrode setup [13] with CC as the current carrying electrode and Ref as the reference electrode enables monopolar recordings under the measurement electrodes M_1_, M_2_, and M_3_. For the first 6 out of 17 subjects, the three measurement electrodes were placed horizontally along the forehead as shown in variant A. For the remaining 11 out of 17 subjects, the measurement electrodes were placed vertically along the forehead above the nose as shown in variant B.

The electrodes were placed in a three-electrode configuration according to Fig. 1, as it allows for monopolar measurements. A saline solution bath, in which the test subjects placed one foot, was used as the current carrying (CC) electrode. The reference (Ref) electrode, which is a prewired Ag/AgCl electrode, initially covered with solid hydrogel (Type: Kendall 1050NPSM, active area of 5.05 cm^2^), was placed on top of the foot, that was not covered by saline solution. The three measurement electrodes M_1_, M_2_, and M_3_ (prewired dry Ag/AgCl electrodes from Wuhan Greentek PTY LTD with an active area of 0.283 cm^2^) were taped to the skin.

For six subjects, the electrodes were placed horizontally with approx. 2 cm distance to each other and about 2 cm above the eyebrow as shown in Fig. 1 (see variant A), and for 11 subjects the electrodes were placed vertically above the nose with approx. 1 cm distance to each other (see variant B). The measurement electrodes were wiped with ethanol before and after use. All electrodes (including CC and Ref) were placed on the same side of the body that was chosen randomly. However, in Variant B there was no difference between left and right hand side for the placement of the M-electrodes.

Two different types of electrical measurements were performed in this study. A small signal alternating current (AC) admittance measurement was done with an applied sinusoidal voltage with frequency of 20 Hz and amplitude of 100 mV. It was possible to obtain the small signal AC conductance (which is the real part of the admittance) via the lock-in technique [17], see also [18]. The chosen frequency and amplitude makes this measurement linear (see [9]), and thus the applied voltage does not affect the conductance of the skin. This measurement had a sample rate of around 2 samples per second and was used for reading of the stored information. A non-linear electrical measurement technique (the applied voltage affects the conductance of the skin [9, 14]) was used to write the information into the skin. Here periodic DC voltage pulses that lasted 30 seconds, followed by 10 seconds of 0 V were used. The pulse height was –1 V and +1 V for the first 6 subjects and –0.5 V and +0.5 V for the 11 test subjects that followed after (to avoid saturation of the measurement instrumentation and reduce the currents through the test subjects). Sample rate for reading and writing was 12.5 samples per second. A non-linear electrical test measurement with a sinusoidal voltage with frequency of 0.05 Hz and amplitude 1 V was done, in which the pinched hysteresis loop was recorded for two periods (similar to the recordings in [9]). Within this test measurement, the electrodes were pushed a little bit. If the recording under an electrode changed significantly or if there was only noise in the recording, then the electrode was retaped. After galvanic contact to the skin under all three measurement electrodes was assured, the actual experiment was going to begin.

### Experiment

**Fig. 2:**
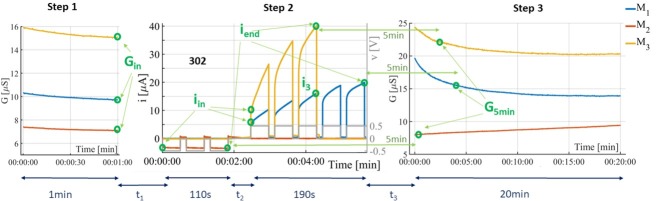
Results of one whole recording of test subject “A” before exercise. Parameterization and durations are visualized. The initial conductance was measured for around one minute in step 1. In step 2, DC voltage pulses were applied to change the conductance of the skin (non-linear measurements). The procedure was different for the two different numbers (302 and 123); this example shows the procedure for number 302. An example measurement for 123 can be seen in Fig. 3. In step 3, the AC conductance was measured for 20 minutes. The start of each measurement was done manually within the software and thus the durations *t_1_*, *t_2_*, and *t_3_* between measurements varied slightly among the test sessions. The initial conductance, here defined as the last conductance value within step 1 is labeled as *G_in_*. Within step 2, *i_in_* and *i_end_* are the current values at the beginning and the end of each DC voltage pulse series. For the further analysis, the conductance values at specific times (3, 5, 10, 15, and 20 minutes) related to the time of the last DC pulse (which is different for each electrode) were used. In the plot to the right (step 3), only the conductance values at minute five, *G_5min_*, are indicated.

**Fig. 3:**
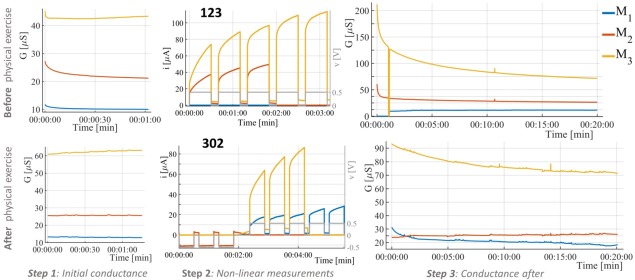
Recordings of test subject “B” before (top plots, number 123 in this case) and after (bottom plots, number 302 in this case) physical exercise shown for the skin under all three measurement electrodes (M_1_, M_2_, and M_3_). DC voltage pulses were applied (middle) and small signal conductance was measured before (left) and after (right) for around 1 minute, and 20 minutes, respectively. The drop of the conductance to zero under M_3_ in the 20 minutes conductance measurement is an artifact.

The initial small signal conductance was measured for around 1 minute (see Fig. 2), followed by DC voltage pulses to write the information into the skin and finally the small signal conductance was measured for 20 minutes after the last DC voltage pulse was applied. This experimental procedure was done two times (before and after physical exercise) for each test subject within one test session. It was randomly chosen after each initial conductance measurement, whether number 123 or number 302 was written to the skin. The subjects randomly picked a sheet of paper, containing one or the other number. The procedure for writing the information into the skin was dependent on the drawn number (see Figs. 2 and 3). If the number was 123, the M_1_ electrode was disconnected from the instrumentation before the positive DC pulses were applied. After the third DC pulse was completed (after 110 seconds), the electrode M_2_ was also disconnected from the instrumentation and two extra DC pulses were applied to electrode M_3_ only. If the number was 302, M_1_ and M_3_ were disconnected from the instrumentation and three negative pulses were applied to M_2_. After that, M_2_ was disconnected from the instrumentation while M_1_ and M_3_ were connected to it and positive pulses were applied. After the third positive pulse, M_3_ was disconnected, and two additional pulses were applied to M_1_ only.

After the fifth positive DC pulse in both variants, all electrodes were connected to the instrumentation again before the small signal conductance measurement started.

The physical exercise between measurement runs consisted of 20 Burpees. However, most of the test subjects did not feel any sweating from it and did some extra physical exercises like 10 pushups or squats of their free choice. The Ref and the M electrodes were kept attached to the skin of the test subjects during the exercise.

## Parameterization and statistical analysis

Ratios of the measured small signal conductance values rather than the absolute conductance values were used for the statistical evaluation. These ratios are expressed by the conductance values at specific time points in step 3 related to the initial conductance *G_in_* in step 1 (see Fig. 2).

For example, the conductance ratio that relates the conductance at 5 minutes after the last DC pulse1In case of number 123, no DC voltage pulses were applied to M_1_. The time for the conductance under M_1_ is related to the last applied DC pulse as it is for M_3_. (occurs at different times under each electrode) to *G_in_* is expressed by


(3) $$G_{\text{Ratio }}(t=5\min)=\frac{G_{5\min}}{G_{in}}.$$

Statistical evaluation of G_Ratio_ at different time points was done separately for the three different skin sites by the aid of SigmaPlot (Version 14.5). The repeated measures ANOVA on ranks (Friedman test) and the Tukey test for pairwise comparison were used for non-normal or heteroskedastic data.

Another parameter of interest is the increase or decrease in current caused by the applied voltage within step 2, expressed as


(4) $$i_{\text{Ratio }}=\frac{i_{end}}{i_{\text{in }}},$$

with *i_end_* as the current at the end of each DC voltage series related to the initial current, *i_in_* (see Fig. 2). Three DC voltage pulses were applied in case of states 0 and 2, and 5 DC pulses in case of state 3 and *i_end_* is related to the 3^rd^ and 5^th^ pulse, respectively as indicated in Fig. 2. The current ratio


(5) $$i_{\text{Ratio }5\text{ to }3}=\frac{i_{\text{end }}}{i_{3}}$$

is only obtained in case of state 3, and gives information about how much the current changed from the 3^rd^ to the 5^th^ DC pulse.

## Results

In total, 34 data sets were collected (17 test subjects, 2 recordings per test subject). It was randomized whether 123 or 302 was written to the skin and the test subjects did physical exercise between the two recordings. In total, 19 measurements were done with number 123 and 13 measurements were done with number 3022For two test subjects, 3 instead of 5 pulses were erroneously applied to M_1_, resulting in number 202.. In table 1 in the appendix, it is shown how many test subjects had which number before and after exercise.

The recordings of one test subject before and after exercise (with numbers 123 and 302 in this case) are shown in Fig. 3. The recordings of another test subject are shown, in Fig. 6 in the supplemental information and the recordings of all subjects are stored and available on Figshare (see also section “Data availability” below). As for most of the test subjects, the initial conductance of the skin under each of the measurement electrodes was quite different to each other (see also Fig. 5 in the supplemental information). This is true for both electrode positioning variants (A and B, see Fig. 1). In step 2, the DC voltage pulses were sufficient to cause electro-osmosis in the sweat ducts and the resulting current and thus the DC conductance were affected. The subject shown in Fig. 3, experienced during the measurement with number 123 an increase in current under the electrode M_3_ from 25.7 μA to 113.8 μA (after the 5^th^ pulse) which results in a ratio (*i_ratio_*) of 4.43. Under M_2_, the increase was from 13.3 μA to 49.5 μA (after the third pulse, *i_ratio_* = 3.7). No DC pulses were applied to M_1_ (state 1).

**Fig. 4:**
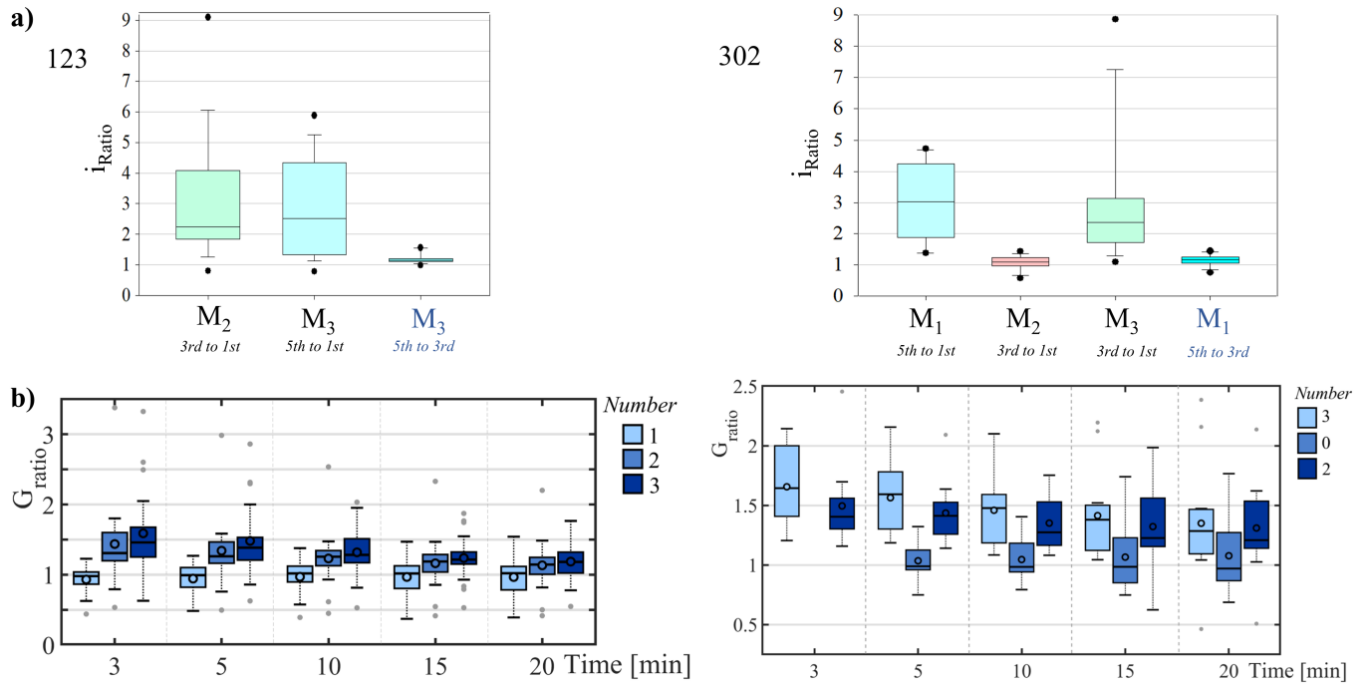
Boxplots based on all measurements with number 123 (left, N=19) and number 302 (right, N=13). **a)** Boxplots of *i_Ratio_*. In case of number 123, no DC voltage pulses were applied to M_1_, three positive pulses were applied to M_2_ and 5 to M_3_. In case of number 302, 5 positive DC pulses were applied to M_1_, 3 negative pulses were applied to M_2_ and 3 positive pulses were applied to M_3_. **b)** Boxplots of *G_ratio_* over time for the three different M electrodes. States 1, 2, and 3 (left plot, N=19) were stored in the skin under the electrodes M_1_, M_2_, and M_3_ respectively. Friedman test and pairwise comparison (Tukey test) show that there are significant differences between state 2 and state 1 and also between states 3 and 1 but not between states 2 and 3 at all time points (see also table 2 in the supplemental information). States 3, 0, and 2 (right plot, N=13) were stored in the skin under the electrodes M_1_, M_2_, and M_3_ respectively. Similar as for number 123, Friedman test and pairwise comparison (Tukey test) show that there are significant differences between state 2 and state 0 and also between states 3 and 0 but not between states 2 and 3 at all time points. There is no data for M_2_ (state 0) at minute 3 since 190 seconds of positive DC voltage pulses followed before the small signal conductance was conducted (see Fig. 2).

The measurement after physical exercise was done with number 302 for this subject (see Fig. 3 bottom plots). The resulting current ratios, *i_ratio,_* were 0.94 in case of M_2_ (from - 11.19 μA to -10.57 μA), 3.37 in case of M_1_ (from 8.45 μA to 28.45 μA) and 2.45 in case of M_3_ (from 35.19 μA to 86.32 μA). The negative DC voltage pulses had almost no effect on the conductance (*i_ratio_* close to 1) of the skin under M_2_, which applies to all recordings with 302 (see Boxplots of *i_ratio_* over all subjects in Fig. 4a). In contrast, the positive DC voltage pulses had a clear impact on the current (and thus the conductance), which can be seen by *i_ratio_* in Fig. 4a. In case of the measurement with number 123 there was a median increase in current by 2.25 times under electrode M_2_ (state 2) and 2.51 under M_3_ (state 3). The difference between states 2 and 3 as defined is the number of DC pulses applied (3 vs. 5). However, it shows that the median current under M_3_ after the fifth pulse is only 1.15 times greater than that after the third pulse, thus the difference is small. In case of the measurements with 302, the median of *i_ratio_* was slightly higher than for the measurements 123 under the corresponding electrodes and might be explained by the small sample sizes (N=19, and N=13).

In step 3, the small signal conductance and thus *G_ratio_* (see Fig. 4b) of states 2 and 3 decrease (fast decrease in the beginning that slows down) over the recorded time. This is similar for all subjects. The conductance of state 0 increased slightly. In case of the measurements with number 123, it can be shown by within group comparisons (see also Table 2 in the supplementary information) that there are significant differences (p-value <0.05) at all time points between state 1 and state 2 and also between state 1 and state 3. However, there is no significant difference between states 2 and 3 at any time point. Similar results for the measurements with number 302. At any time point, there are significant differences between state 0 and state 2, between state 0 and state 3 but there are no significant differences between states 2 and 3.

**Fig. 5:**
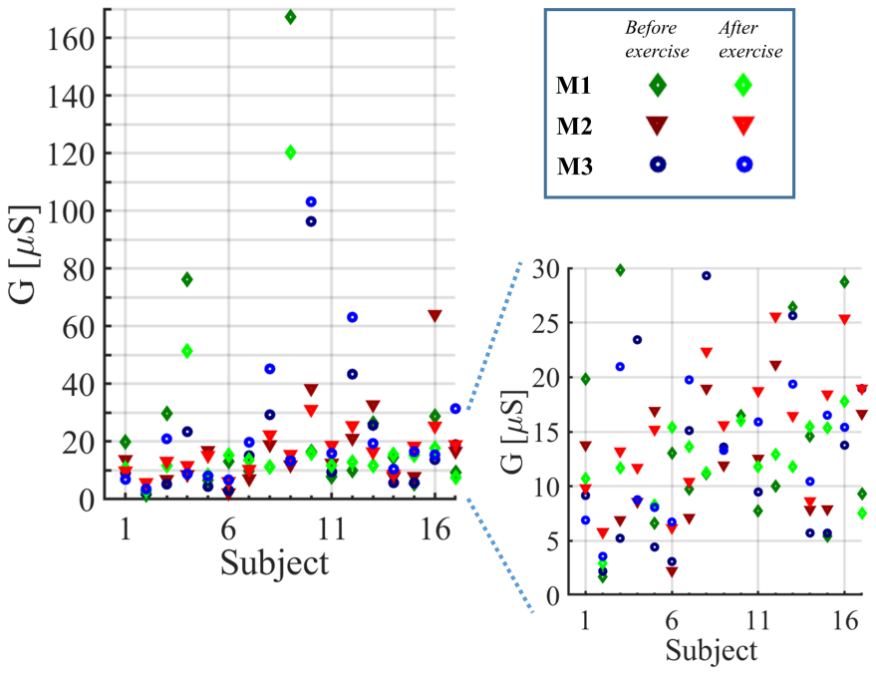
Initial conductance values, *G_in_*, under each electrode (M_1_, M_2_, and M_3_) shown for all 17 test subjects before and after exercise. The plot in the lower right corner is a zoomed in version of the plot to the left.

## Discussion

In this study we tried to write information into human skin. With DC pulses we affected the conductance of the skin by changing the sweat level within the sweat ducts and measured the small signal conductance for 20 minutes afterwards to see for how long the written information (i.e. the change in conductance) can be obtained. Since there were large variations in skin conductance between skin sites and test subjects, we introduced *G_ratio_*, which relates the measured conductance under each electrode to its baseline (initial conductance, *G_in_*). We defined 4 possible states under each electrode dependent on the DC voltage pulses that were applied in step 2 (3 negative pulses for state 0, state 1 as baseline state without application of any pulses, 3 and 5 positive pulses for states 2, and 3, respectively). Positive DC voltage pulses cause electro-osmosis of the sweat towards the skin surface and thus increase the conductance of the skin while negative pulses move the sweat towards inner skin layers.

Number 123 that was written to the skin via three different measurement electrodes M_1_, M_2_, and M_3_ enabled the comparison between the baseline state and the states 2 and 3. There is no significant difference between states 2 and 3, which may be explained by the very little increase in current from pulse 3 to pulse 5. However, states 2 and 3 can be distinguished from state 1 even after 20 minutes meaning that a binary information (baseline and activated state) can be stored for at least 20 minutes in human skin. If the sweat does not reach the skin surface in the first place, a movement of the sweat towards inner skin layers has almost no effect on the skin conductance, as it could be seen when the negative DC pulses were applied. Therefore, it seems that there cannot be a distinction between an inactivated state (0) and the baseline state (1). However, direct comparison is needed and the results may be different if negative DC voltage pulses with a large pulse height would be applied. As a summary, binary information storage (instead of 4 distinct states) is possible for at least 20 minutes.

It may be possible to achieve more states that are distinguishable by choosing durations and pulse heights of the DC voltage pulses that are different to the ones chosen here. We reduced the pulse height from 1 V to 0.5 V after the first 6 subjects since two of these obtained currents larger than 180 μA. This was done for electrical safety and to avoid saturation of the measurement instrumentation. The changes in current with pulse height of 0.5 V were smaller but still sufficient to achieve the activated state (state 2 or 3). Smaller electrodes (than the ones used here) may allow for more than two states since the applied voltages could be higher (since the current is lower due to decreased area) and changes in conductance would be more specific to single sweat ducts and not subject to averaging effects.

Two recordings were done per test subjects and physical exercise in between was introduced to somehow neutralize the stored information from the first recording. However, the physical exercise was too little to cause sweating and longer exercise sessions should be included in the test protocol to see a significant effect on the conductance. In any way, it was possible to write and read information for the second time (recording after physical exercise).

It was surprising that the initial conductance under each of the three electrodes of one subject was quite different to each other. The electrodes were placed close to each other at the same skin site (forehead). After we observed the variations in the conductance in the first six subjects, we changed the electrode placement from variant A to variant B. However, that did not help and there was still a large variation in the initial conductance. There was no clear tendency, i.e. the highest initial conductance was measured under M_1_, M_2_, or M_3_, which was different from subject to subject. Variation in sweating and skin surface may be one reason for the difference in the initial conductance even though the electrodes were close to each other. Variation in electrode contact may be another reason and the use of different types of electrodes (for example, printed Ag/AgCl electrodes) may improve the measurement quality. However, pushing of the electrodes in the initial test measurement usually affected the measured current only slightly, if at all. Instead of placing the electrodes always at the same position, it would be interesting for future experiments to change the position of the electrodes slightly until skin sites of equal initial conductance are found.

Here we wrote information into human skin by changing the sweat level due to electro-osmosis. However, the sweat level changes naturally, for example due to thermal sweating, as well. Some subjects (4 out of 17) even showed electrodermal activity (EDA) [19] (see Fig. 6 in the supplemental information) at the forehead. The natural sweating has an effect on the conductance and thus the stored information. The conductance during the initial measurement was more constant after the physical exercise. A longer resting time after electrode placement may lead to a more stable baseline of the conductance.

The writing of the information into skin (i.e. introducing electro-osmosis to the sweat in the ducts) can be done with any low frequency AC or DC electrical stimulus with a large enough amplitude. The DC voltage pulses were chosen as a continuation of the work in [14].

From this study it is now known that it is possible to increase the level of sweat in the ducts by an applied voltage and that there is still a higher sweat level observable after 20 minutes. This could be used as an initial treatment of the skin to increase the quality of electro-dermal activity (EDA)

measurements. Furthermore, three electrodes were used in this study to store information underneath. If instead, for example, an electrode array with 10 × 10 electrodes would be used, the information can be more complex, and it seems likely that a binary picture (black and white pixels) can be stored into the skin.

## Figures and Tables

**Table 1:** Possible combinations of applied numbers before and after exercise and obtained number of test subjects (N) per combination.

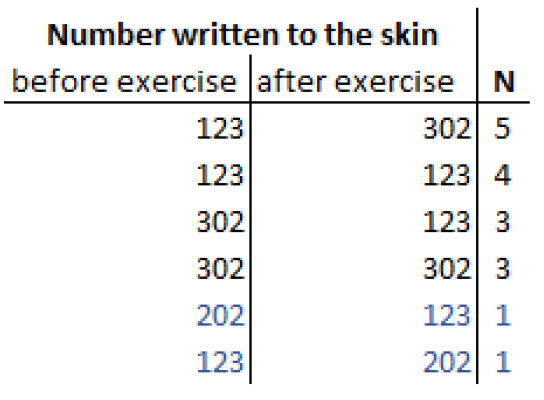

**Table 2:** Statistical evaluation of *G_ratio_(t)* at time points 3, 5, 10, 15 and 20 minutes over all recordings with number 123 (top, N=19) and number 302 (bottom, N=13). Median and 25% and 75% percentiles of *G_ratio_(t)* are presented, followed by the results from Friedman test and the results from the pairwise comparison (Tukey test). Within group comparisons of the measurements with number 123 show that there are significant differences (see P-values) between states 3 and 1 and also between states 2 and 1 but not within states 2 and 3 (P-values > 0.05). This is valid for all picked times. Within group comparisons of the measurements with number 302 show that there are significant differences between states 3 and 0 and also between states 2 and 0 but not within states 2 and 3, which is similar to the measurements with number 123.

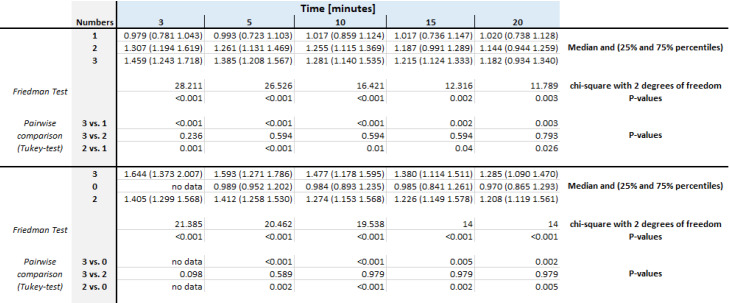

**Fig. 6:**
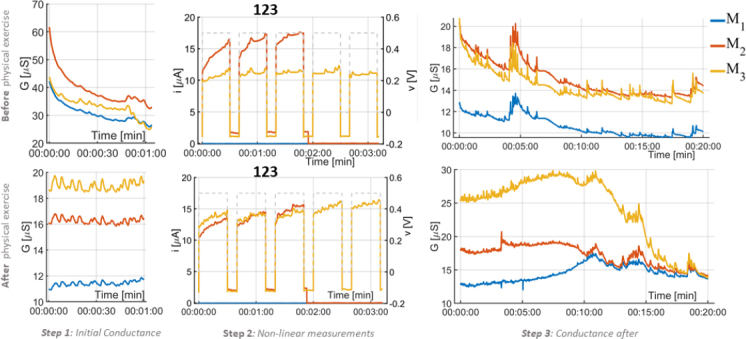
Recordings of test subject “C” before (top plots) and after physical exercise (bottom plots) shown for the skin under all three measurement electrodes (M_1_, M_2_, and M_3_). For this subject, both measurements were done with number 123. This subject was electrodermal active at the forehead.
